# Isolation and Whole-Genome Sequencing of Four Antibiotic-Producing Pseudomonas Strains

**DOI:** 10.1128/mra.00005-22

**Published:** 2022-06-06

**Authors:** Audrey L. E. Armstrong, Kyle Cole, Emily Collins, Jarret M. Easterday, Lauren Feddersen, Brianna Fleury, Lauren E. Johnson, Ankush F. Kecht, Logan H. McClure, Joselyn M. Molinar, Anjali S. Patel, Ria Patel, Jackson R. Rapala, Arunima Salomi, Amari A. Stepter, Michal Wrobel, Loralyn M. Cozy

**Affiliations:** a Department of Biology, Illinois Wesleyan University, Bloomington, Illinois, USA; University of Arizona

## Abstract

Here, we report the isolation, whole-genome sequencing, and annotation of four novel Pseudomonas isolates. We also evaluate the biosynthetic potential of each genome.

## ANNOUNCEMENT

Pseudomonas is a Gram-negative, rod-shaped, polar flagellated bacterial genus with more than 140 identified species (1). Within this genus, there are many species that inhabit a diverse range of environments, resulting in their multifaceted metabolic capacities and adaptation abilities in changing environments. Here, we report the draft genomes of four novel Pseudomonas isolates that exhibit high antibiotic activity.

Soil samples were collected from a variety of locations (Table 1). One gram of each sample was resuspended in 10 mL of sterile saline, diluted serially, and plated on Reasoner’s 2 agar (R2A) (2) hardened medium plates at 25°C for 48 h. Once diluted, 50 colonies were isolated, and individual colonies were screened for antibiotic activity by patching onto lawns of other bacteria, followed by incubation at 25°C for 48 h. The strains described here, LF19, LM20, JR33AA, and KCA11, were selected because they produced zones of inhibition against lawns of several bacterial species (Table 1). The 16S rRNA gene PCR product was sequenced from each strain using the primer set 27F (5′-AGR GTT TGA TYM TGG CTC AG-3′) and 1492R (5′-GGY TAC CTT GTT ACG ACT T-3′), with 55°C annealing and 30 s of extension. Using NCBI BLAST (3), it was determined that the isolates belong to the Pseudomonas genus.

**TABLE 1:** Sampling locations, accession numbers, sequencing features, and genomic characteristics of the Pseudomonas strains

Isolate	Location^*a*^	SRA accession no.	Assembly accession no.	GenBank accession no.	No. of reads	Read length (bp)	No. of contigs	*N*_50_ (bp)	Avg coverage (×)	Size (bp)	GC content (%)	No. of genes	No. of proteins	Antibiotic activity^*b*^
JR33AA	47°37′47″N, 116°46′5″W	SRR17071229	GCF_021378985.1	JAJSAW000000000	4,285,246	146	65	324,782	230	5,407,927	62.02	6,001	5,031	Bs, Sa, Ec, Ab, Pa, Ea
KCA11	40°30′55″N, 88°59′26″W	SRR17071230	GCF_021378935.1	JAJSDJ000000000	4,432,406	151	63	309,066	231	5,554,878	63.15	5,318	5,250	Bs, Sa, Ec, Ab, Pa, Ea
LF19	40°5′51″N, 88°19′9″W	SRR17071231	GCF_021378965.1	JAJSDK000000000	3,550,179	146	76	247,141	177	5,811,296	62.60	5,388	5,322	Bs, Sa, Ab
LM20	40°49′6″N, 89°29′18″W	SRR17071232	GCF_021378925.1	JAJSDL000000000	3,533,723	146	60	316,867	183	5,596,202	63.08	5,295	5,227	Bs, Sa, Pa

a Coordinates (latitude and longitude) of the soil collection site.

b Ab, Acinetobacter baylyi; Bs, Bacillus subtilis; Ea, Enterobacter aerogenes; Ec, Escherichia coli; Pa, Pseudomonas aeruginosa; Sa, Staphylococcus aureus.

**FIG 1:**
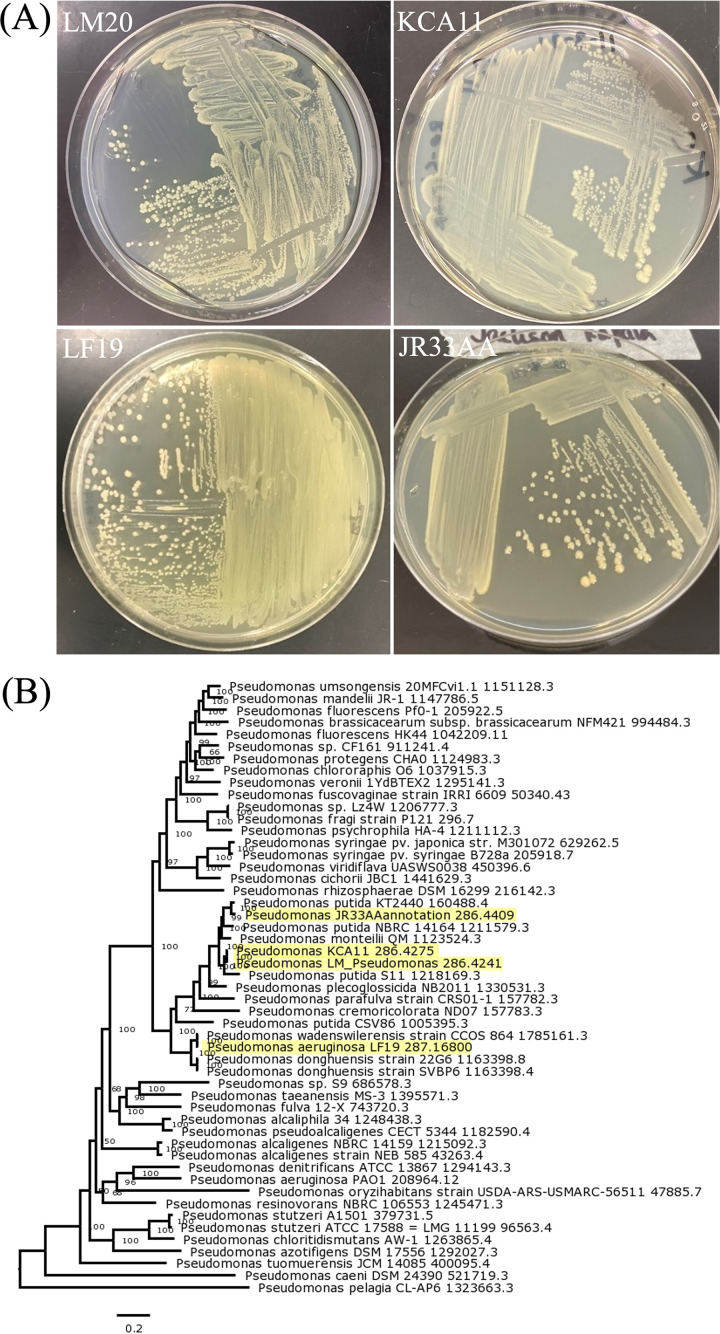
Colony morphology and phylogenetic relatedness. (A) R2A streak plates of each strain as indicated. (B) Phylogenetic tree generated using the Codon Tree pipeline in PATRIC with default parameters, with an input of 51 good-quality genomes. A total of 23,327 amino acids and 69,981 nucleotides were represented by 100 single-copy genes. The best tree was determined with 100 rounds of RAxML bootstrapping. Isolates LF19, LM20, JR33AA, and KCA11 are highlighted in yellow.

Axenic cultures grown on R2A were sent to the Microbial Genome Sequencing Center (Pittsburgh, PA) for DNA isolation using the Qiagen DNeasy blood and tissue kit and whole-genome sequencing (Fig. 1A). Sequence libraries were prepared with a small-volume tagmentation protocol using the Nextera DNA library preparation kit (Illumina, San Diego, CA, USA). Barcodes and adapters were attached, and libraries were amplified using the KAPA HiFi library amplification kit (4). Paired-end libraries were subsequently sequenced on the Illumina NextSeq 550 platform. FastQC v0.11.9 (5, 6) was used to verify the quality of the reads. These reads were then assembled *de novo* in PATRIC v3.6.12 (7) using the Unicycler v0.4.8 program (8). The assembly included polishing using two Pilon iterations (9) and examination using QUAST v5.0.2 (10). Read number and length information can be found in Table 1. All programs related to genome assembly were run with default parameters. Annotation was performed in PATRIC v3.6.12 using the RASTtk pipeline (11) with default bacterial parameters, using Pseudomonas as a taxonomic guide (Table 1).

Taxonomic relationships between the four isolates and other known Pseudomonas species were evaluated using the Codon Tree pipeline (12–15) in PATRIC v3.6.12 (7) in combination with average nucleotide identity (ANI) calculations using the Kostas Lab ANI Calculator v1.0 (16, 17), both run with default parameters. These calculations revealed that LM20 and KCA11 shared 99.5% nucleotide identity, while the remaining relationships all ranged between 82.5% and 89.3%. Additionally, isolates LM20, KCA11, and JR33AA all group with Pseudomonas putida strains, while LF19 is most related to Pseudomonas wadenswilerensis and Pseudomonas donghuensis strains (Fig. 1B).

Genome mining for antibacterial compounds using antiSMASH v6.0.1 (18), with the relaxed strictness setting, provided evidence that each of the four strains contains biosynthetic gene clusters (BGCs) potentially encoding secondary metabolites. The strain with the most predicted BGCs is LF19, with 13, and the strain with the least is LM20, with 8. KCA11 and JR33AA are both predicted to possess 10 BGCs. These strains add to the genomic data available for the Pseudomonas genus, supporting further investigation into its biosynthetic potential.

### Data availability.

This whole-genome shotgun project was deposited in GenBank under BioProject PRJNA784595. GenBank assembly and Sequence Read Archive (SRA) accession numbers are presented in Table 1.
